# Acute Myeloid Leukemia with Co-mutated *ASXL1* and *SRSF2* Exhibits Monocytic Differentiation and has a Mutational Profile Overlapping with Chronic Myelomonocytic Leukemia

**DOI:** 10.1097/HS9.0000000000000292

**Published:** 2019-09-11

**Authors:** Steven M. Johnson, Daniel R. Richardson, Jonathan Galeotti, Sonia Esparza, Anqi Zhu, Yuri Fedoriw, Karen E. Weck, Matthew C. Foster, Catherine C. Coombs, Joshua F. Zeidner, Nathan D. Montgomery

**Affiliations:** 1 Department of Pathology and Laboratory Medicine, The University of North Carolina School of Medicine, Chapel Hill, NC, USA; 2 Lineberger Comprehensive Cancer Center, The University of North Carolina School of Medicine, Chapel Hill, NC; 3 Division of Hematology and Oncology, Department of Medicine, The University of North Carolina School of Medicine, Chapel Hill, NC, USA; 4 The Cecil G. Sheps Center for Health Services Research, University of North Carolina at Chapel Hill, Chapel Hill, NC, USA; 5 Department of Biostatistics, Gillings School of Global Public Health, The University of North Carolina, Chapel Hill, NC, USA.

In recent years, the genetic profile of acute myeloid leukemia (AML) has been extensively characterized, and multiple prognostically distinct subgroups have emerged. One subgroup arising in elderly patients harbors somatic mutations in RNA splicing factor genes (eg, *SF3B1, SRSF2*) and/or chromatin-related genes (eg, *ASXL1*). Mutations in these gene categories frequently co-occur in AML, in which case they result in additive adverse prognostic effects. In particular, AML with *ASXL1* and *SRSF2* mutations (*ASXL1*^mut^*SRSF2*^mut^, “co-mutated” AML) has been associated with a dismal prognosis, similar to AML with high-risk cytogenetic features.^1^ However, there is very little published literature regarding additional clinical or pathologic features of this AML subgroup.

After *TET2*, *ASXL1* and *SRSF2* are the most commonly mutated genes in chronic myelomonocytic leukemia (CMML),^2–4^ a myelodysplastic (MDS)/myeloproliferative neoplasm (MPN) clinically characterized by peripheral monocytosis and frequent transformation to acute leukemia.^2,5^ To date, the immunophenotype of *ASXL1*/*SRSF2* co-mutated AML has not been characterized. However, in AML arising from antecedent CMML, leukemic blasts tend to retain monocytic differentiation.^6^ As such, we speculated that *ASXL1/SRSF2* co-mutation may also drive monocytic differentiation in AML, potentially in addition to other clinicopathologic similarities with CMML.

To address this possibility, we retrospectively identified adult AML patients (≥18 years) with *ASXL1/SRSF2* co-mutation identified in diagnostic myeloid sequencing panel analysis performed at our institution over a 3-year time period (April, 2015-April, 2018). One-hundred and fifty consecutive non-co-mutated AML (ie, AML with *ASXL1*^wt^*SRSF2*^wt^, *ASXL1*^mut^*SRSF2*^wt^, or *ASXL1*^wt^*SRSF2*^mut^) from April, 2016 to April, 2018 were collected for comparison (see Supplemental Methods, Supplemental Digital Content). Concomitant hematopathology reports were reviewed to confirm diagnoses in all cases. Patients with sequencing data available only from recurrent/relapsed disease were excluded, as were cases of acute promyelocytic leukemia.

Clinical sequencing and flow cytometric analysis (FCA) methods are detailed in the Supplemental Methods (Supplemental Digital Content). Briefly, next generation sequencing was performed on DNA collected from diagnostic bone marrow or peripheral blood samples using the Illumina® TruSight Myeloid 54-gene sequencing panel (Illumina, San Diego, CA). Cases where immunophenotypic FCA was performed on blood or bone marrow aspirate specimens within three months of sequencing without intervening curative therapy were included.

Sixteen patients with *ASXL1*/*SRSF2* co-mutated AML were identified during the study period. All patients with *ASXL1*/*SRSF2* co-mutated AML were over 50 years old at diagnosis and significantly older than patients with non-co-mutated AML (median age 69 years versus 63 years, *P* = 0.042, Table 1). AML with myelodysplasia-related changes (MRC), based on the current World Health Organization (WHO) criteria, was seen in 8 (50%) patients, five of which arose secondarily in a known five of which arose secondarily in a known MDS with excess blasts (2016 WHO Classification).^7^ No patients carried a prior documented CMML diagnosis, although three of five patients (60%) classified as de novo AML who had available complete blood counts prior to diagnosis had an occult peripheral blood monocytosis, documented between 7 and 36 months before AML onset (Table S1). This observation raises the possibility that a subset of these AML cases may have evolved from unrecognized CMML.

**Table 1:**
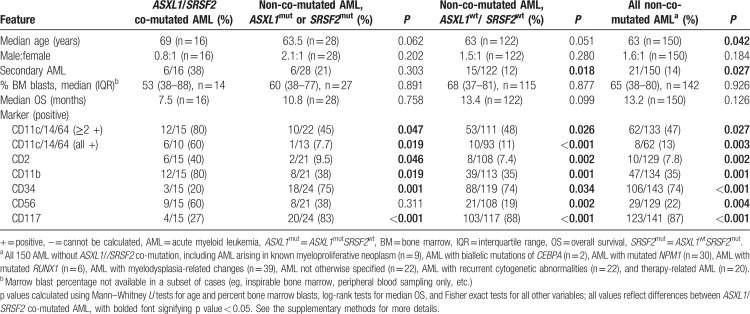
Clinicopathologic features of *ASXL1/SRSF2* co-mutated AML vs non-co-mutated AML

All but one *ASXL1/SRSF2* co-mutated AML showed overt evidence of monocytic differentiation. Specifically, 80% (12/15) of *ASXL1/SRSF2* co-mutated AML cases with available FCA data expressed 2 or more of the monocytic lineage-defining markers CD11c, CD14, or CD64, compared to 47% of non-co-mutated AML (Table 1 and Fig. S1). Of the 3 *ASXL1/SRSF2* co-mutated AML cases that did not express ≥2 monocytic markers, 1 patient had a peripheral monocytosis at diagnosis (4.1×10^9^/L, 10% of peripheral white blood cells), and the leukemic blasts of another patient expressed one monocytic marker and gated in the monocyte region by FCA (as determined by CD45 expression and side scatter, data not shown), supporting monocytic differentiation.

Several additional immunophenotypic features provided further evidence of monocytic differentiation (Fig. S1). CD56 and CD2, which are frequently aberrantly expressed on monocytes in CMML,^8^ were more commonly expressed in *ASXL1/SRSF2* co-mutated AML than in non-co-mutated cases (60% and 40% vs 22% and 8%, respectively; *P* = 0.004 and 0.002, respectively). In addition, the myeloblast markers CD34 and CD117, which are not typically expressed in monocytic precursors, were less commonly expressed in *ASXL1/SRSF2* co-mutated AML than in non-co-mutated AML (20% and 27% vs 74% and 87%, respectively; *P* < 0.001 for both comparisons). A representative flow cytometry plot from a patient with *ASXL1/SRSF2* co-mutated AML is shown in Figure S2.

Having observed that *ASXL1/SRSF2* co-mutated AML tends to exhibit evidence of monocytic differentiation, we next sought to determine if this association was dependent on co-mutation or whether similar trends existed for cases of AML with mutation in either *ASXL1* or *SRSF2*. Compared to AML cases with either *ASXL1* or *SRSF2* mutation but not both (n = 28, collectively), *ASXL1/SRSF2* co-mutated AML was significantly more likely to express 2 or more monocytic markers (80% vs 45%; *P* = 0.047), more likely to express aberrant CD2 and CD56 (40% and 60% versus 10% and 38%, respectively; *P* = 0.046 and 0.311, respectively), and significantly less likely to express the myeloblast markers CD34 and CD117 (20% and 27% vs 75% and 83%, respectively; *P* = 0.001 and <0.001, respectively) (see Table 1 and Fig. S1). These results support more prominent monocytic differentiation in *ASXL1/SRSF2* co-mutated cases.

Next, we evaluated the mutation landscape of *ASXL1/SRSF2* co-mutated AML, which also differed from control cases. For all analyses, bulk sequencing was performed without cell sorting, and inferences were made about clonal relationships and disease ontogeny using comparison of variant allele fractions (VAFs). In all cases, *SRSF2* mutations were identified at a VAF of ∼50%, suggesting the presence of a heterozygous activating mutation in the majority of cells. By comparison, VAF of *ASXL1* mutation was more variable, ranging from 13% to 47%, but was >25% in 13/16 co-mutated cases (Table S1 and Fig. S3). These results suggest that the majority of cells harbored concomitant *ASXL1* and *SRSF2* mutations in most cases.

*TET2*, the most commonly mutated gene in CMML, was more likely to be mutated in *ASXL1/SRSF2* co-mutated cases than in non-co-mutated cases (63% vs 19%; *P* < 0.001, Table 2). In addition, 25% of *ASXL1/SRSF2* co-mutated AML had more than one *TET2* mutation, compared to only 5% of non-co-mutated cases (*P* = 0.018). Other mutations more commonly seen in *ASXL1*/*/SRSF2* co-mutated AML compared to non-co-mutated AML included *STAG2* and *IDH1/IDH2* (*P* < 0.001 and *P* = 0.027, respectively). Notably, mutations in these genes are often enriched for in AML with mutated chromatin-related or RNA splicing factor genes.^1^ No other genes on our 54-gene panel were mutated at statistically significant differences between cohorts (data not shown). However, after excluding the presence of *ASXL1* and *SRSF2* mutations, we noted that *ASXL1/SRSF2* co-mutated AML harbored more total variants (median 3 per patient) than *ASXL1*^wt^*SRSF2*^wt^ AML and all non-co-mutated AML (median 2 per patient, respectively, *P* = 0.023 and 0.043).

**Table 2:**
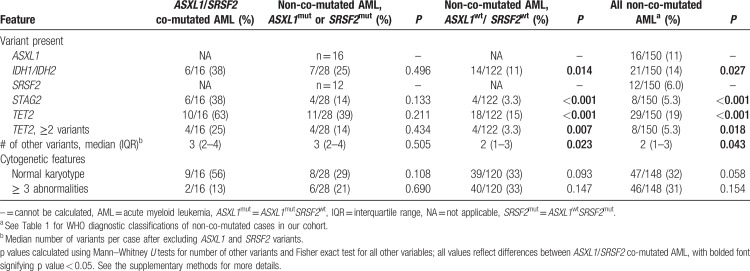
Genetic features of *ASXL1/SRSF2* co-mutated AML vs non-co-mutated AML

In terms of cytogenetic features, half of *ASXL1/SRSF2* co-mutated AML cases had a normal karyotype at diagnosis, and none harbored AML-defining recurrent cytogenetic abnormalities (Table S1).^1^ Three patients (19%) with *ASXL1/SRSF2* co-mutated AML had trisomy 8, the most common cytogenetic abnormality identified in CMML.^9^ Although cytogenetic abnormalities were uncommon in *ASXL1/SRSF2* co-mutated AML, morphologic dysplasia in 2 or more myeloid lineages was seen in 8/11 (73%) evaluable *ASXL1/SRSF2* co-mutated AML cases, including 4/6 (67%) cases with history of a prior myeloid neoplasm and 4/5 (80%) de novo cases (Table S1).

Given the relatively small sample sizes in this cohort, it is not possible to make definitive conclusions regarding patient outcomes in *ASXL1/SRSF2* co-mutated AML. However, overall survival (OS) was generally poor, as nearly all patients died within 2 years of AML diagnosis (Table S1). The apparently poor outcomes associated with this genotype may be related to disease ontogeny, as *ASXL1/SRSF2* co-mutation occurred more often in patients with a prior myeloid neoplasm (secondary AML, Table 1) compared to patients with *ASXL1*^wt^*SRSF2*^wt^ AML (*P* = 0.018). These results are in keeping with findings from earlier studies which identified mutational signatures of secondary AML.^1,10^ Finally, while we did confirm a trend towards inferior OS in patients with co-mutated *ASXL1/SRSF2* compared to *ASXL1*^wt^*SRSF2*^wt^ AML patients (*P* = 0.099), no pairwise comparisons of OS between study groups reached statistical significance (Table 1).

The association between certain co-occurring mutations and distinct monocytic phenotypes is well-established. For instance, monocytic cytomorphology is frequently seen in *NPM1*-mutated AML with *FLT3*-internal tandem duplication.^11^ Moreover, in patients with chronic myeloid neoplasms with dysplasia, *SRSF2* and *TET2* mutations have been frequently associated with monocytosis and myelomonocytic phenotype, particularly when both are mutated concurrently.^12^ For instance, Awada et al^13^ showed that the presence of 2 or more *TET2* mutations with VAF sum >55% most commonly occur in MDS/MPN, particularly CMML and other myeloid neoplasms associated with monocytosis and marrow myeloid dysplasia. *SRSF2* mutations also frequently co-occurred with multiple *TET2* mutations, further supporting a potential relationship between *SRSF2* mutation and monocytic differentiation.^13^

In conclusion, we show that *ASXL1/SRSF2* co-mutated AML shows evidence of monocytic differentiation and has genetic overlap with CMML, including the frequent presence of *TET2* mutations. In addition, *ASXL1/SRSF2* co-mutated AML frequently arises secondarily in patients with antecedent myeloid malignancy, and even when these mutations apparently occur “de novo,” patients may have unrecognized peripheral monocytosis prior to leukemic onset. Whether *ASXL1/SRSF2* co-mutation is a biological driver of monocytic differentiation remains uncertain. In addition, it will be important to confirm these observations in larger, multi-institutional studies. Nonetheless, our findings raise the possibility that this subset of AML may often arise as a secondary AML from an occult CMML-like MDS or MDS/MPN even in the absence of a known prior myeloid neoplasm.

## Supplementary Material

Supplemental Digital Content
