# Supplementary material for: Acute Myeloid Leukemia with Co-mutated ASXL1 and SRSF2 Exhibits Monocytic Differentiation and has a Mutational Profile Overlapping with Chronic Myelomonocytic Leukemia
Source: Hemasphere. 2019 Sep 11;3(5):e292. doi: 10.1097/HS9.0000000000000292 (PMC6919468; doi:10.1097/HS9.0000000000000292)
Supplement: Supplemental Digital Content [file hs9-3-e292-s001.pdf]

Supplemental Methods:

Case identification and statistical analysis:

Results from all diagnostic myeloid next generation sequencing panels (see below for methods) performed on clinical samples at our institution were reviewed to identify potential cases of *ASXL1*/*SRSF2* co-mutated AML. In total, we identified 16 *ASXL1*/*SRSF2* co-mutated AML since the date of origination of our clinical assay (April 1, 2015). Of note, our comparison cohort consists of 150 consecutive non-co-mutated AML identified after April 15, 2016, as performing archival pathology report searches prior to this date are technically challenging due to our institution's transition to a new laboratory information system at this time.

Descriptive statistics were reported to characterize the patient population. Median ages and ratios of males to female were reported to describe demographics. Medians and interquartile ranges (IQR) were reported to describe the distributions of bone marrow blast counts (%) and number of variants per case. Medians were reported for overall survival in months. All categorical variables were reported as numbers or proportions. Patients were grouped by their mutational status, and all variables were tabulated for *ASXL1/SRSF2* co-mutated AMLs and then compared to non-co-mutated AMLs. Mann-Whitney U tests were used to compare median counts between groups. Fisher's exact tests were used to test the association of dichotomic variables between groups. Log-rank tests were used to compare median overall survival between groups. Statistical analyses were performed using Prism 8 (GraphPad Software, Inc., La Jolla, CA). A two-sided alpha value <0.05 was considered significant.

Immunophenotypic flow cytometric analysis:

Expression levels of informative markers were extracted from diagnostic hematopathology reports, with ≥20% expression on leukemic cells considered positive. CD11c, CD14, and CD64 represent monocytic markers as described in the primary text. Cases where flow cytometric analysis was performed at an outside facility (3/16, 19% of *ASXL1*/*SRSF2* co-mutated cases; 21/150, 14% of non-co-mutated cases) were included, provided sequencing analysis was completed at our institution on a time-matched specimen prior to curative therapy. For in-house cases, four- or eight-color flow cytometry was performed on clinical peripheral blood or bone marrow aspirate specimens using a panel of antibodies to detect the following informative markers in most cases: CD2, CD3, CD4, CD5, CD7, CD8, CD10, CD11b, CD11c, CD13, CD14, CD15, CD19, CD20, CD33, CD34, CD45, CD56, CD64, CD71, CD117, HLA-DR, and kappa and lambda light chains. Data were acquired using a FACS Canto flow cytometer and analyzed using Cell Quest software (Becton Dickinson, San Jose, CA).

Cytogenetic analysis:

Conventional cytogenetic studies were performed as part of clinical testing according to standard methods.[1] Briefly, chromosome preparations were G-banded using trypsin and Wright's stain, and karyotypes were interpreted and described according to the International System for Human Cytogenetic Nomenclature. When tabulating cytogenetic aberrations, a single/reciprocal structural or numerical abnormality, was counted as 1 abnormality. Karyotypes with ≥3 abnormalities were considered complex.

Next generation sequencing:

DNA was extracted from clinical blood or bone marrow aspirate samples using an established laboratory method utilizing Qiagen EZ1 (Qiagen, Hilden, Germany). DNA samples were quantified by fluorometry using a Qubit (Thermo Fisher Scientific, Middletown, VA, USA) prior to library preparation, as a DNA input of 50 ng (in a maximum volume of 15 uL) is required for library preparation. Indexed sequencing libraries were then prepared according to the manufacturer's instructions (Illumina, San Diego, CA, USA). Please refer to the Illumina website for the complete gene panel list and coverage (https://www.illumina.com/products/by-type/clinical-research-products/trusight-myeloid.html). Pooled libraries were sequenced on an Illumina MiSeq, and variant annotation was performed using the Illumina Variant Studio application. With the exception of the one *ASXL1* variant described below, all known or likely pathogenic variants in all genes were included if the VAF was ≥ 5%. Of note, *ASXL1* c.1934dupG [p.Gly646fs] is a known potential technical artifact in the Illumina platform when occurring at a low variant allele fraction (VAF) (~5%), while those identified at a higher VAF likely represent true pathogenic variants.[1,2] We have independently validated this variant in our clinical testing as a true variant at VAFs greater than 10% by confirmation with an orthogonal method (Sanger sequencing). As such, cases in which this variant was present at VAF >10% are included in this study.

Parallel mutational analysis of a normal germline sample is not routinely performed at the time of a new AML diagnosis, unless a patient is suspected to have a myeloid neoplasm with germ line predisposition. However, all variants are rigorously annotated, including comparison to both germline databases (e.g. ExAC, gnomAD, ClinVar) and databases of somatic mutations previously

reported in cancer (COSMIC). All mutations included in our mutational analyses represent ASCO/AMP/CAP Tier I and II variants; likely benign variants and variants of unknown significance were both excluded.[3]

Table S1. Clinicopathologic characteristics of AML with *ASXL1*/*SRSF2* co-mutations.

				Prior Monocytosis[a]								
**#**	**Age/ Sex**	**Prior MN[b]**	**Diagnosis (WHO)**	**+/−**	**Abs, rel**	**Interval (mo)**	**BM dysplasia[c] (lineages)**	***ASXL1* variant (VAF, %)**	***SRSF2* variant (VAF, %)**	**Other pathogenic/likely pathogenic variants (VAF, %)**	**Karyotypic features**	**OS[d] (mo)**
1	66/M	+	Arising in MPN (s-AML)	−	N/A	N/A	1 of 3	c.1934dupG (37)	p.P95H (49)	*TET2* (44), *SETBP1* (46)	+21	>12.4
2	81/F	+	MRC (s-AML)	−	N/A	N/A	3 of 3	c.1934dupG (19)	p.P95H (54)	*DNMT3A* (24), *TET2* (89)	+1, add(1)(p12)	2.3
3	74/F	+	MRC (s-AML)	−	N/A	N/A	3 of 3	p.R693* (39)	p.P95H (43)	*RUNX1* (38), *STAG2* (37), *TET2* (59)	Normal	8.9
4	68/F	+	MRC (s-AML)	−	N/A	N/A	3 of 3	c.1934dupG (27)	p.P95H (42)	*CEBPA* (37), *IDH2* (37), *STAG2* (28)	Normal	2.6
5	81/F	+	MRC (s-AML)	−	N/A	N/A	NA	c.1934dupG (27)	p.P95H (39)	*TET2* (7)	del(12)(p11.2p13)	26.6
6	71/M	+	MRC (s-AML)	−	N/A	N/A	3 of 3	c.1934dupG (37)	p.P95H (59)	*BCOR* (17), *BCOR* (73), *NRAS* (36), *RUNX1* (47), *STAG2* (92), *TET2* (44)	Normal	11.0
7	59/F	+	t-AML	+	1.8, 15.3%	15	1 of 3	c.2172_2177 delinsAG (13)	p.P95_R102 del (54)	*FLT3* TKD (24), *NRAS* (14), *NRAS* (6), *NRAS* (5), *STAG2* (20), *TET2* (46), *TET2* (44)	+1, del(4)(q32), i(13)(q10)	6.1
8	65/M	−	MRC	+	1.1, 16.9%	36	2 of 3	c.2298delC (47)	p.P95H (59)	*CEBPA* (12), *IDH2* (49), *KRAS* (14), *NRAS* (33)	+8	>16.1
9	71/M	−	MRC	−	N/A	N/A	2 of 3	c.1934dupG (39)	p.P95H (58)	*IDH1* (48), *JAK2* (47), *RUNX1* (53)	+8	5.5
10	71/M	−	MRC	+	1.7, 11.7%	7	1 of 2	c.1934dupG (40)	p.P95H (49)	*JAK2* (8), *TET2* (86), *TP53* (47)	+8, (10;22)(q22;q13), +19, del(20)(q11.2)	1.5
11	67/M	−	NOS	NA	N/A	N/A	3 of 3	p.R1068* (41)	p.P95_R102 del (56)	*IDH2* (47)	Normal	15.5
12	70/F	−	NOS	+	1.8, 13.7%	8	2 of 3	c.1888_1910 del23 (23)	P95H (56)	*ETV6* (17), *RUNX1* (20), *TET2* (50), *TET2* (46)	Normal	0.6
13	69/F	−	NOS	−	N/A	N/A	NA	c.1934dupG (37)	p.P95R (48)	*NRAS* (14), *STAG2* (44), *TET2* (49), *TET2* (41)	Normal	0.07
14	69/M	−	NOS	NA	N/A	N/A	N/A	p.G679* (41)	p.P95R (45)	*FLT3* TKD (29), *IDH1* (41), *STAG2* (54)	Normal	4.5
15	52/F	−	*NPM1*	NA	N/A	N/A	N/A	c.1934dupG (28)	p.P95H (47)	*NPM1* (32), *TET2* (45), *TET2* (43), *TET2* (6)	Normal	>11.1
16	64/F	−	*NPM1*	NA	N/A	N/A	N/A	p.Y591* (41)	p.P95H (46)	*IDH1* (37), *NPM1* (30)	Normal	>11.7

AML: acute myeloid leukemia; MN: myeloid neoplasm; +: present; −: absent; Abs, Rel: peripheral blood absolute (cells/µL) and relative monocytosis (% of white blood cells), respectively. WHO: Diagnosis by World Health Organization classification, revised 4th edition (Arising in MPN: AML arising in patient's prior myeloproliferative neoplasm; MRC: AML with myelodysplasia-related changes; s-AML: secondary AML (excluding therapy-related AML); t-AML: therapy-related AML; TKD: tyrosine kinase domain mutation; NOS: AML, not otherwise specified; *NPM1*: AML with mutated *NPM1*); BM: bone marrow; VAF: variant allele fraction (%); N/A: not applicable; OS: overall survival (months); NA: not available.

[a] Peripheral blood monocytosis prior to AML diagnosis. Interval represents duration between peripheral blood monocytosis and AML diagnosis. Patient #8 had two CBCs one month apart, both of which had absolute and relative monocytosis. The values reported for this patient represent his latest CBC, 8 months prior to AML diagnosis. Patients #10 and #12 had only a single available CBC for review, so evaluation of persistence was not possible.

[b] Patient #1 had a reported *JAK2* p.V617F-mutated MPN. Patients #2-6 had reported histories of *de novo* MDS diagnoses (refractory anemia with excess blasts, RAEB-1 or RAEB-2) prior to AML progression. For patients #3 and #5, sequencing was done at time of RAEB diagnosis, with progression to AML 4 and 9 weeks after initial sequencing, respectively. The variants for these two patients listed here represent those identified at time of RAEB diagnosis; no additional sequencing was performed at time of progression to AML. Patient #7 had a history of marginal zone lymphoma treated with rituximab, cyclophosphamide, vincristine sulfate, and prednisone, and developed a therapy-related RAEB-1 prior to AML progression.

[c] Background marrow dysplasia identified in the bone marrow aspirate in patients who did not have >90% marrow blasts (i.e. excluding patients #14-16). Patients #6 and #13 were diagnosed by peripheral blood testing only, as no BM biopsy was performed for clinical reasons. No megakaryocytes were identified in patient #9's diagnostic aspirate.

[d] Overall survival was calculated from date of AML diagnosis.

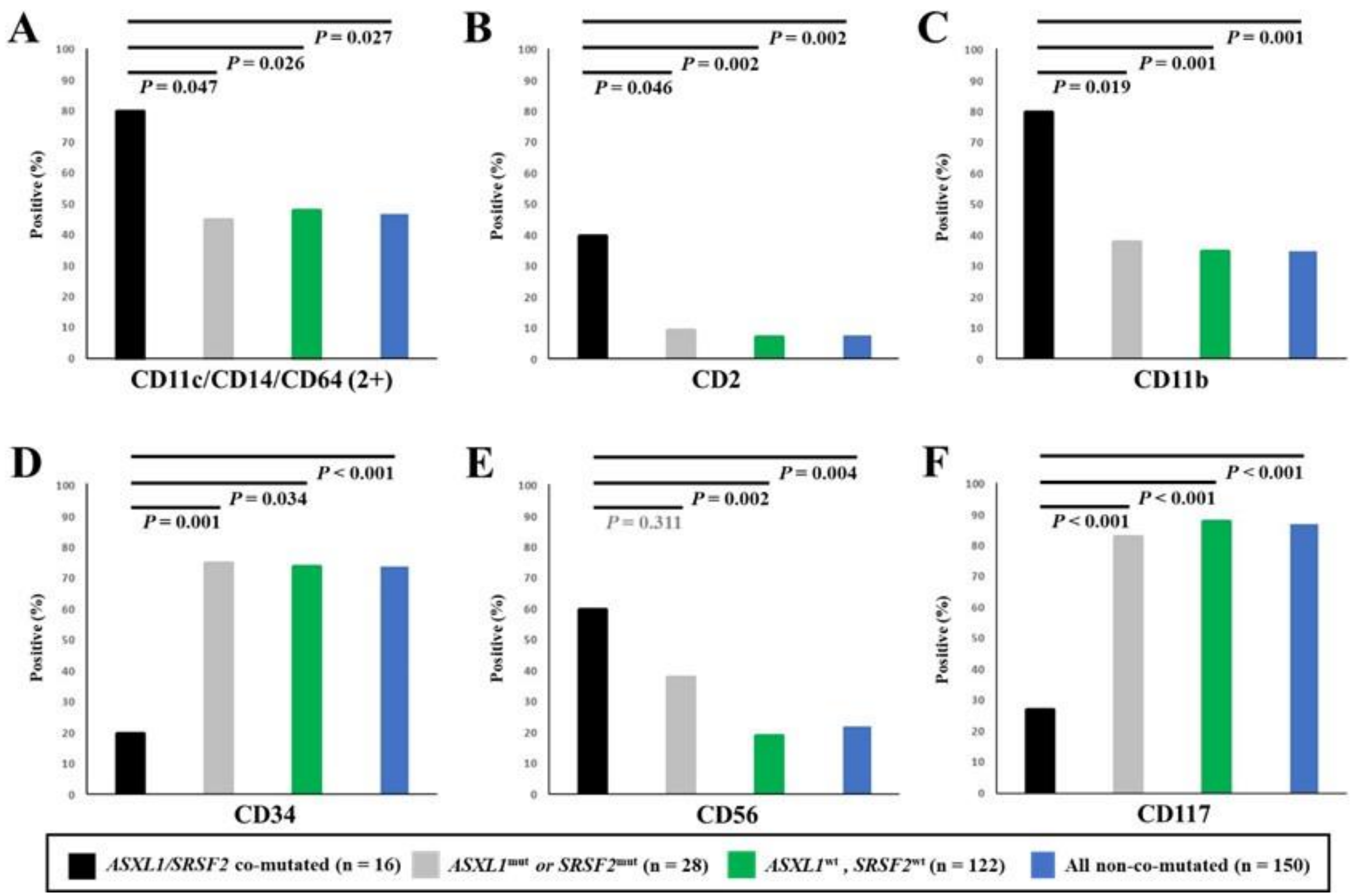

Figure S1. Immunophenotypic features of *ASXL1*/*SRSF2* co-mutated AMLs vs. non-co-mutated AMLs. For all markers the percentage of positive cases is shown for *ASXL1*/*SRSF2* co-mutated AML (n = 16, black bars), AML with *ASXL1* or *SRSF2* mutated but not both (n = 28, gray bars), *ASXL1*^{wt}/*SRSF2*^{wt} AML (n = 122, green bars), and all non-co-mutated AML (n = 150, blue bars). Markers analyzed are (A) cases with 2 of CD11c, CD14, and/or CD64 positive, (B) CD2, (C) CD11b, (D) CD34, (E) CD56, and (F) CD117. *P* values calculated using Fisher's exact tests; all values reflect differences between *ASXL1*/*SRSF2* co-mutated AML, with bolded font signifying *p* value < 0.05.

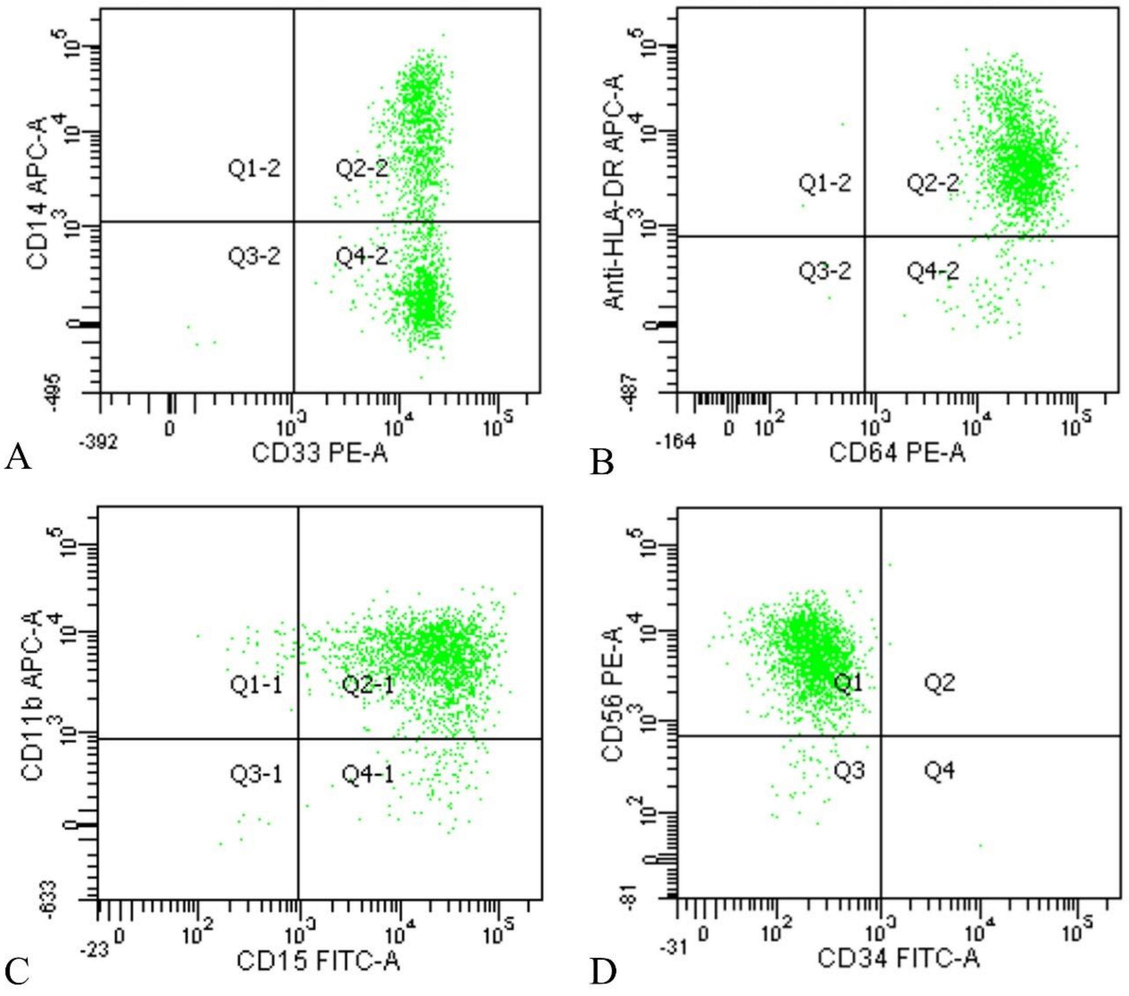

Figure S2: Immunophenotypic flow cytometric analysis from a representative patient (#14) with *ASXL1*/*SRSF2* co-mutated acute myeloid leukemia. The leukemic cells in this case were (A) positive for CD33, predominantly positive for CD14, (B) positive for CD64, HLA-DR, (C) CD15, CD11b, and (D) were negative for CD34, while showing aberrant CD56 expression.

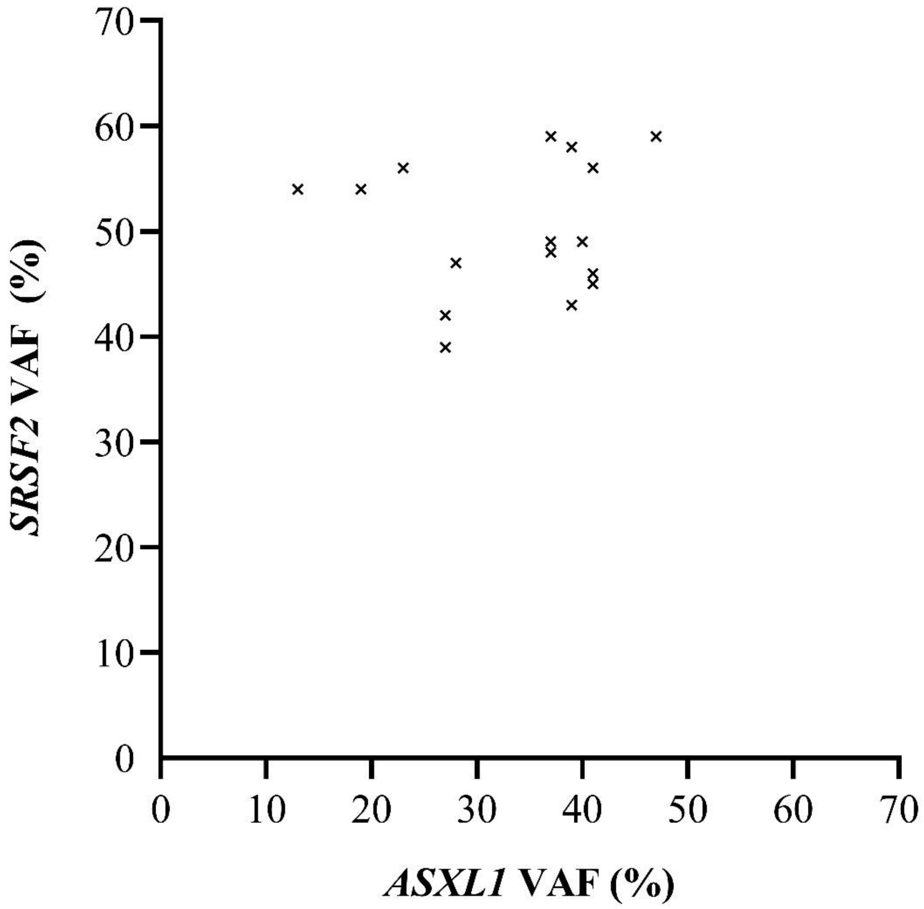

Figure S3: Scatter plot of variant allele fractions (VAF) for *ASXL1* and *SRSF2* mutations from each patient (one per point) in our cohort with *ASXL1*/*SRSF2* co-mutated acute myeloid leukemia.

Supplemental References:

1. Thomas M, Sukhai MA, Zhang T, et al. Integration of technical, bioinformatic, and variant assessment approaches in the validation of a targeted next-generation sequencing panel for myeloid malignancies. *Arch Pathol Lab Med*. 2017;141(6):759-775.
2. Yannakou CK, Jones K, McBean M, et al. ASXL1 c.1934dup;p.Gly646Trpfs*12—a true somatic alteration requiring a new approach. *Blood Cancer J*. 2017;7(12):656.
3. Li MM, Datto M, Duncavage EJ, et al. Standards and Guidelines for the Interpretation and Reporting of Sequence Variants in Cancer: A Joint Consensus Recommendation of the Association for Molecular Pathology, American Society for Clinical Oncology, and College of American Pathologists. *J Mol Diagn*. 2017;19(1):4-23.
